# DEVELOPING A CONCEPTUAL MODEL TO ASSESS ACTIVE ENGAGEMENT WITHIN A DEMENTIA CAREGIVER INTERVENTION

**DOI:** 10.1093/geroni/igad104.0464

**Published:** 2023-12-21

**Authors:** Sarah Neller, Moroni Fernandez Cajavilca, Julene Johnson, Lee Ellington, Jacqueline Eaton

**Affiliations:** University of Tennessee, Knoxville, Knoxville, Tennessee, United States; New York University, New York City, New York, United States; University of California San Francisco, San Francisco, California, United States; University of Utah, Salt Lake City, Utah, United States; University of Utah, Salt Lake City, Utah, United States

## Abstract

Active engagement is identified as an effective component of psychoeducational interventions for caregivers of persons living with dementia. There are many approaches to active engagement; however, limited assessment of this component within interventions reduces optimization and standardization. To fill this gap and increase transparency, we aimed to strengthen active engagement by developing Enhancing Active Caregiver Training (EnACT), an arts-based intervention designed to help caregivers address the behavioral symptoms of dementia. The intervention uses multi-sensory activities, including interacting with caregiver-informed vignettes, to foster engagement and process caregiving experiences. We partnered with caregivers of persons living with dementia (n=9) to iteratively refine EnACT through six focus groups. The purpose of this presentation is to report the process we took to identify how intervention participants actively engaged with EnACT. Focus group data were analyzed using process coding to identify the patterns of participant engagement with 17 intervention activities. The process codes were mapped to create a diagram of participant engagement for each activity. These diagrams were used to develop the EnACT Cycle of Engagement, a conceptual model illustrating how participants engaged with the intervention. The three-step model illustrates that: 1) antecedents (personal and intervention) contribute to 2) interaction with EnACT through participation, contribution, and application, which leads to 3) EnACT’s hypothesized proximal outcomes (capacity to adapt and appraisal of caregiving demands) and distal outcomes (perceived stress and caregiver well-being). The EnACT engagement model will be tested in the forthcoming randomized control trial assessing feasibility and acceptability.

